# The hen and the egg question in atopic dermatitis: allergy or eczema comes first

**DOI:** 10.1186/s40733-023-00090-2

**Published:** 2023-02-10

**Authors:** Anastasiia Allenova, Razvigor Darlenski

**Affiliations:** 1 Laboratory of Immune-Mediated Skin Diseases, I.M. Sechenov First Moscow State Medical University (Sechenov University), Moscow, Russia; 2 Medical Research, and Education Center, M.V. Lomonosov Moscow State University, Moscow, Russia; 3 Department of Dermatology and Venereology, Acibadem City Clinic Tokuda Hospital Sofia, 51B Nikola Vaptsarov Blvd., 1407 Sofia, Bulgaria; 4 Department of Dermatology and Venereology, Trakia University-Stara Zagora, Stara Zagora, Bulgaria

**Keywords:** Eczema, Atopy, Asthma, Atopic march, Treatment

## Abstract

Atopic dermatitis (AD) as a chronic inflammatory systemic condition is far more than skin deep. Co-morbidities such as asthma and allergic rhinitis as well as the psychological impact influence seriously the quality of life of the patients. Recent studies have shown that only 10% of atopic patients undergo full manifestation of the atopic march, while 40% demonstrate concomitant food allergy. Exposure to food allergens in the environment causes sensitization and food allergy through the disruption of the skin barrier, as in AD. Food allergy and AD are closely related. While not all AD patients have a food allergy, 20–40% of children with moderate to severe AD will have an IgE-mediated food allergy. It is known that they may coexist but it is unclear if food allergy worsens the course of AD. Experimental, clinical, and epidemiological studies have provided evidence of the primary role of an epidermal barrier defect in the development of sensitization to environmental allergens and that this process occurs in the damaged skin barrier rather than the gastrointestinal or respiratory tract. There is strong evidence for a connection between early AD onset and the development of other allergic diseases later in life.

## Introduction

Being a chronic and systemic inflammatory condition atopic dermatitis (AD) affects not only the skin. A significant deterioration in the quality of life of patients with AD is determined by the presence of comorbidities, such as asthma and allergic rhinitis, as well as psychological factors. In addition to skin eczema, the complex of atopic diseases also includes asthma, allergic rhinoconjunctivitis, food allergy, and eosinophilic esophagitis [1, 2]. The unifying factor in this group is type 2 inflammatory response (Th2 helper cells pathway), with the central role of interleukin (IL)-4 and IL-13 [3]. This complex of pathologies in most cases in the life of patients with atopy is characterized by a consistent course, and the transition of the pathological process from one organ and system to another is called an «atopic march». Recent studies have shown that only 10% of atopic patients undergo full manifestation of the atopic march, while 40% demonstrate concomitant food allergy [4]. Exposure to food allergens in the environment causes sensitization and food allergy through the disruption of the skin barrier, as in AD. However, immune tolerance can be achieved by early oral exposure to food allergens [5]. The current vision of AD suggests two main directions in the disease pathophysiology: 1) disorders in the epidermal barrier as a result of a genetically determined absence or violation of the filaggrin synthesis — a protein involved in the construction of the stratum corneum and components of the natural moisturizing factor [6]; 2) immunological disorders with a predominance of the Th2-immune response with the key cytokines IL-1, IL-4, IL-5, IL-13, IL-31 but the list of candidate cytokines and immune mediators of inflammation is constantly expanding. The question that worries the scientific community for a long time is what exactly is the root cause of the atopic complex: allergy or skin ezcema. In the last decade, much evidence has been provided supporting the idea that a defect in the epidermal barrier plays the primary role in the initiation of sensitization to allergens from the environment and that this process occurs through the damaged skin barrier rather than through the gastrointestinal tract or respiratory system [7]. This fact has also been confirmed by large cohort epidemiological studies of the age distribution of the atopic spectrum diseases: a relationship has been shown between the early onset of AD and the development of asthma and allergic rhinitis at school age [8]. The risk appears to be even higher in children with persistent early-onset AD phenotype [9]. Clinical studies also support the concept of atopic march from early skin barrier dysfunction to the development of food sensitization and clinical food allergy. Regarding inhaled allergens and AD, they are strongly related and often coexist. In certain patients with AD and concomitant inhalation allergy, the skin condition was worsened during the provocation season. These data are also confirmed by positive results in AD patients receiving immunotherapy inducing tolerance to aeroallergens.

### Epidemiology data

AD affects 15–20% of children and 2–10% of adults [1, 10]. In recent decades, the incidence has been steadily increasing. In 60% of patients AD develops in the first year of life, and in 85% of patients’ skin changes develop by the age of 5. In severe cases, the disease persists after puberty. According to recent data, about one-third of AD cases in adults appear much later - after age twenty. Most AD patients have a mild form of the disease, but about 10% have serious clinical manifestations. In adults, this percentage is higher than in children [2]. It is estimated that about 50% of patients with AD also have other allergic problems during the first year of life. Among them, 85% show symptoms of other allergic diseases within the first 5 years of life. In about 70% of patients, AD resolves spontaneously up to puberty.

### Genetic predisposition to atopic dermatitis

AD is undoubtedly characterized by strong genetic effects. About 77% of identical and 15% of fraternal twins develop AD [1, 2, 4, 10]. Intact healthy skin is an important physical and immunological barrier against the entry of allergens and microbes from the environment into the body. Defects of the skin barrier can be caused by a variety of factors, including defects in terminal epithelial differentiation, such as deficiency of filaggrin (FLG), an important structural protein of the stratum corneum, deficiency of antimicrobial peptides, changes in the intercellular lipids of the stratum corneum, microbiome dysbiosis, and impaired immune regulation [7].

Genetic defects encoding skin barrier proteins, as well as abnormalities in lipid production or tight junctions, contribute to the epidermal barrier dysfunction that is a specific feature of AD. Mutations with loss of filaggrin function are associated with an earlier onset and a more stable AD phenotype. It has been also reported that polymorphism in the thymic stromal lymphopoietin (TSLP) gene and its receptor, as well as dysregulation of genes involved in the metabolism of epidermal lipids, are associated with an increased risk of AD, its resistance, and food allergy. Other mutations of the skin barrier, including the SPINK5 gene and сorneodesmosin mutations, are also associated with AD and the development of food allergy [7, 11]. Newborns with increased transepidermal water loss (TEWL) in the first week of life had an increased risk of developing AD at 12 months of age [12]. These genetic factors, which play a significant role in the onset of AD, support the point of view that damage to the skin barrier may present at a very early age, even before the clinical onset of AD.

### Skin barrier dysfunction leads to epicutaneous sensitivity and food allergy

Currently, there is increasing evidence supporting the view that allergic skin sensitization occurs more readily through the damaged skin barrier. Several studies performed in mice describe immunological pathways involved in the progression from epicutaneous sensitization to food allergy [13]. Now it is clear that the mechanism of food allergy is through the epicutaneous sensitization, while the oral exposure leads to tolerance [13, 14]. Clinical studies also support the concept of atopic march from early skin barrier dysfunction to the development of food sensitization and clinical food allergy. It has been shown that an increase in TEWL on the 2nd day of life is a predictor of food allergy at the age of two [15]. Newborns with a filaggrin defect have an increased risk of developing AD and asthma. People with AD and filaggrin defects also have an increased risk of pollen allergy.

### Allergy to respiratory and food haptens in AD patients

As is commonly known, food allergy and AD are closely related. While not all AD patients have a food allergy, 20–40% of children with moderate to severe AD will have an IgE-mediated food allergy [2, 7]. It is known that they may coexist but it is unclear if food allergy worsens the course of AD. Some studies show that in case of a positive egg allergy test patient’s condition can improve if they eliminate eggs from their diet. This creates some cause for concern since eliminating the allergen from the diet can interfere with the development of oral tolerance in patients. According to guidelines, children under 5 years old with moderate to severe AD may be tested for food allergies to milk, eggs, peanuts, wheat, and soy if at least one of the following conditions is met: 1) the child has a reliable history of an immediate reaction (e.g., urticaria, swelling, itching, sneezing, coughing, wheezing, vomiting, and low blood pressure) after swallowing certain food; 2) the child has persistent AD despite optimized therapy. Food allergy develops in only around 30% of AD patients [16]. However, many speculations about cow’s milk protein allergy (CMPA) exist. AD and CMPA diagnostics are not equivalent. The only reliable method for confirming food allergy - it’s an elimination provocation test with the appropriate food. All other laboratory tests (skin prick test, in vitro immunoglobulin E test, etc.) are of low diagnostic and prognostic value. Several randomized controlled trials have shown that early administration of allergenic foods such as peanuts or eggs to high-risk infants with severe AD or food sensitization may reduce the risk of developing peanut or egg allergy [12, 15]. Regarding inhaled allergens and AD, they are strongly related and often coexist. Common allergens are pollen, mites, dogs, and cats. Several studies showed that AD patients who had a positive skin reaction to dust mites and suffered a bronchial inhalation provocation test with dust mites, developed skin symptoms in 50% of cases after this test, mainly in areas of the body where eczema usually occurs. All of these patients also have decreased pulmonary function. Other authors do not find a correlation between the inhalation provocation test and AD aggravation. Our clinical observation shows that in some patients with AD and concomitant inhalation allergy, the skin condition worsens during the provocation season. These data are also confirmed by positive results in AD patients receiving immunotherapy inducing tolerance to aeroallergens. Modern progress has led to the development of new drugs that suppress elements of the pathological immune response (mediated by Th2), characteristic of both AD and allergic rhinitis and asthma. Thus, in these patients, such drugs would have a good effect on various comorbidities of the atopic spectrum, and not only on the cutaneous or respiratory manifestations.

### Preventive strategies: practice lessons

Many primary prevention interventions aim to reduce the risk of AD by prophylactically protecting the skin barrier, starting at a very young age in high-risk infants. The generally recommended preventive intervention for the development of asthma and atopic march, namely the use of emollients that restore the skin barrier [1, 6, 7, 17], has recently been challenged [18]. It has been even shown that the use of emollients increases the risk of food allergy development [19]. Whether this is an association or a causal relationship, remains to be elucidated.

Regular use of emollients from the first day after birth reduces the risk of clinical AD and asthma development by 30–55% until 2 years old [1, 6, 7, 17]. There is some evidence that probiotic supplementation during pregnancy or infancy may protect against AD development but there is no proven protection against food allergies or other allergic disorders. There is still insufficient evidence to support the use of other dietary components such as prebiotics, hydrolyzed formulas, or vitamin D supplements for the primary prevention of allergic diseases [2, 7]. Several primary prevention strategies for asthma and allergic rhinitis have also been studied. These include avoiding contact with ticks in early childhood and preventive sublingual immunotherapy in sensitized children. However, no sufficient evidence for the admission of these methods into routine clinical practice has been provided [5, 7]. Secondary prevention of allergic diseases includes interventions in high-risk children or children with a disease, such as AD or sensitization, aimed at preventing progression to the next phase of the atopic march.

## Conclusion

Experimental, clinical, and epidemiological studies have provided evidence of the primary role of an epidermal barrier defect in the development of sensitization to environmental allergens and that this process occurs in the damaged skin barrier rather than the gastrointestinal or respiratory tract. There is strong evidence for a connection between early AD onset and the development of other allergic diseases later in life. Current preventive interventions, such as early regular use of emollients in high-risk infants, should be reappraised and probably the future will disclose the optimal strategy to restore epidermal barrier effect - the primary event in AD.

## Data Availability

Available with the authorship.
